# Biliary stents are forgotten more frequently in elderly patients

**DOI:** 10.3906/sag-2104-108

**Published:** 2021-09-27

**Authors:** Ali Erkan DUMAN, Hasan YILMAZ, Sadettin HÜLAGÜ

**Affiliations:** Division of Gastroenterology, Department of Internal Medicine, Faculty of Medicine, Kocaeli University, Kocaeli, Turkey

**Keywords:** Biliary stents, elderly patients

## Abstract

**Background/aim:**

Plastic biliary stents that remain in situ for more than 12 months, called forgotten biliary stents (FBSs), can cause complications such as cholangitis, stent migration, stent occlusion, and perforation.

**Materials and methods:**

The medical records of patients who underwent ERCP procedures from December 2016 to December 2020 were analysed retrospectively. Data on patient characteristics, indications for ERCP and stenting, stent types, stenting duration, complications, and causes of FBSs were obtained from the hospital’s database.

**Results:**

A total of 48 cases with FBSs were analysed. The mean age (SD) of the patients was 71.23 years (±12.165), the male-to-female ratio was 23/25 (0.92), and the mean stenting duration was 27.12 months (range: 12–84 months). The most common indication for biliary stenting was irretrievable choledochal stones (40/48). Stone formation (79%) and proximal stent migration (26.4%) were the most frequent complications. The patients in the FBS group were significantly older than those from whom stents were removed in a timely manner (71.23 vs. 62.43 years, p < 0.001). Endoscopic treatment was possible in all cases; surgery was not required in any case. The most common cause of FBSs cited by patients was not having been informed about the need for long-term management of their stents (n = 14, 29.2%)

**Conclusion:**

FBSs are potentially problematic particularly in elderly patients. Communication with the patient to remind them of the need for stent management is important for preventing FBSs.

## 1. Introduction

Endoscopic retrograde cholangiopancreatography (ERCP) with endoscopic biliary stenting (EBS), using a plastic or metallic stent, is the standard of care for choledocholithiasis and several other obstructive biliary diseases. Metallic stents are typically used in cases with malignant obstruction of bile ducts, and plastic stents for benign biliary strictures or irretrievable choledochal stones. Plastic stents are not intended for permanent use and should be replaced after 3–6 months [1]. Stents retained for more than 12 months are termed forgotten biliary stents (FBSs), and result in complications such as stent occlusion, migration, cholangitis, and perforation [2,3]. Other complications—such as diarrhoea, haemobilia, and giant stentoliths—are also encountered, albeit rarely [4–6].

Endoscopists performing ERCP frequently encounter FBSs. Most information on FBSs is from case reports; few studies have evaluated FBSs’ complications and management [7–9]. In this retrospective study, we evaluated the incidence, complications, and management of FBSs in patients undergoing ERCP.

## 2. Materials and methods

The medical records of patients who underwent ERCP procedures performed in the Gastroenterology Endoscopy Department of Kocaeli University Medical Faculty from December 2016 to December 2020 were analysed retrospectively. Data on patient characteristics, ERCP and stenting indications, stent types, stenting duration, and complications were obtained from the hospital database. Biliary plastic stents inserted for benign diseases, which remained in situ for more than 12 months, were defined as FBSs. The causes of FBSs were obtained from the medical records and by conducting telephone interviews.

Ethical approval for this study was obtained from the Ethics Committee of Kocaeli University, Faculty of Medicine (approval number: GOKAEK-2020/21.7).

Statistical analysis was performed using SPSS for Windows software (ver. 20.0;IBM Corp., Armonk, NY, USA). The Kolmogorov–Smirnov and Shapiro–Wilk tests were used to assess the normality of the data. Continuous variables are presented as means ± standard deviation or medians (ranges). Categorical variables are shown as counts (percentages). Continuous variables were compared between groups by independent-samples t-test. Associations between categorical variables were examined by chi-squared test. In all analyses, a two-sided p-value <0.05 was indicative of statistical significance.

## 3. Results

A total of 1460 ERCP procedures were performed in 959 patients between December 2016 and December 2020. The mean (SD) age of the patients was 61.04 (17.119) years. Of the patients, 473 were female (49%) and 486 male (51%), and 487 biliary plastic stents and 87 self-expandable metallic stents were placed (Table 1). Forty-eight biliary plastic stents remained in situ for more than 12 months in 44 patients; the mean (SD) age of those patients was 71.23 (12.165) years, and the median stenting duration was 22.5 (12–84) months (Table 1). The most common reason for FBSs reported by patients was not having been informed of the need for long-term stent management (29.2%). Seven patients had undergone cholecystectomy after ERCP and believed that their stent had been removed during the procedure. Six patients were noncompliant, possibly because they believed that their stent was to remain in place permanently (Table 1).

Out of 48 plastic biliary stents, 41 had been placed in our department, and 7 had been placed at other centres. Thirty-four FBSs (one previous) were encountered during ERCP, most of which were symptomatic. A database search identified 14 (3 previous) asymptomatic FBSs; the patients were contacted by telephone. Because of restrictions in place as a result of the ongoing coronavirus disease 2019 (COVID-19) pandemic, those patients could not be called for ERCP and stent removal. Instead, we explained the symptoms of FBSs and instructed them to visit the Endoscopy Department if they experienced any. The patients in the FBS group were significantly older than those from whom biliary stents were removed in a timely manner (71.2 vs. 62.4 years, p < 0.001) (Table 2).

**Figure 1:**
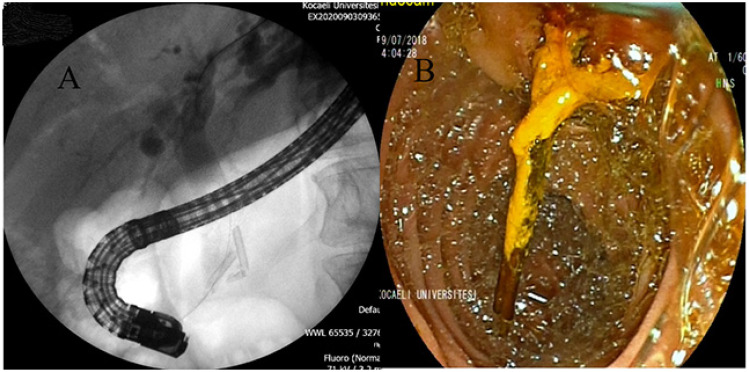
**A**. Fragmented stent in the distal part of the choledochus, 24 months after stent placement. **B**. Stentolith 20 months after stent placement.

**Figure 2:**
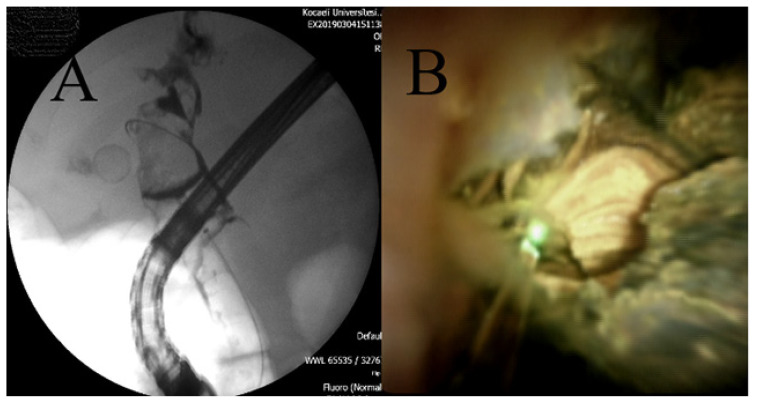
**A**. Giant stone 48 months after stent placement. **B**. Laser lithotripsy of the stone.

The most common indication for biliary stenting was irretrievable choledochal stones, followed by benign biliary strictures and post-cholecystectomy biliary leak. Stone formation was noted in three of the seven patients in whom the initial stenting indication was benign biliary stricture or post-cholecystectomy biliary leak. A stentolith was seen in one case (Figure 1B) and a giant stone in another patient (Figure 2A); the latter was managed by cholangioscopy and laser lithotripsy (Figure 2B). Proximal migration of stents and stent fracture (Figure 1A) were also encountered. Endoscopic treatment was possible in all cases; surgery was not required in any case. Non-migrated stents were easily retrieved using a snare or basket. A new stent was placed in the case of large stones or a biliary stricture. Of the nine proximally migrated stents in our series, four were retrieved endoscopically. We did not consider surgery in the remaining cases because biliary drainage was maintained by exchanging the stent. If endoscopic retrieval during follow-up ERCP was not feasible, cholangioscopy-assisted retrieval would be considered. The stenting indications, clinical presentations, FBS-related complications, and applied endoscopic treatments are given in detail in Table 3.

## 4. Discussion

Plastic biliary stents are used to maintain bile flow in patients with benign biliary diseases, such as irretrievable choledochal stones, benign biliary strictures, or post-cholecystectomy biliary leaks. In this study, the most common indication for biliary stenting was irretrievable common bile duct stones. The mean duration of stenting was 27.12 (range: 12–84) months. In cases of irretrievable choledochal stones, biliary stents may be used as bridge therapy to maintain bile flow and reduce stone size and, thus, facilitate later endoscopic removal [10–12]. In such instances, biliary stents remain in place for 2–6 months before definitive endoscopic therapy. Plastic biliary stents may be used for longer periods in elderly patients and those with contraindications for surgery. Pisello et al. treated 30 high-risk patients with difficult common bile duct stones by permanent stenting; the median follow-up was 38 months. The most frequent late complication is cholangitis, which is managed by stent substitution [13]. In long-standing biliary stents, obstruction by plugs is problematic. Plugs are formed by biliary sludge accumulation and result in bacterial adhesion microbial biofilm growth, which promotes cholangitis [14]. Biliary stents should remain in situ for no more than 3–6 months to prevent cholangitis [1]. Cholangitis was the most frequent finding among the patients with symptomatic FBS in our case series. Even a completely obstructed stent may not disrupt bile flow because of the existence of a passage between the choledochus and the stent [8]. Therefore, FBSs can be asymptomatic for long periods.

The patients in our FBS group were older than those in whom the plastic biliary stents had not been forgotten. This is because elderly people face physical impairment and mental and social problems, leading to increased dependency [15] and hampering access to healthcare services. Therefore, more caution is required to prevent FBS when placing stents in elderly patients.

In most cases, FBSs can be retrieved by endoscopic techniques. However, management of long-term-retained stents is hampered by complications such as stent migration and fragmentation, as well as “giant stentolith” formation. Bacterial colonisation of stents triggers the release of b–glucuronidase, which deconjugates bilirubin glucuronide into calcium bilirubinate crystals. These crystals aggregate on the stent and form a stone cast, i.e. a stentolith [16]. Giant stentoliths must be removed surgically [4,17]. In a prior series, most FBS cases were managed surgically [9], whereas in another surgery management was not needed [8]. The study with a high surgery rate [9] had longer mean and maximum stent patency times (3.53 [range: 1–14] years) compared with those in our study (22.6 ± 12[range: 12–84] months) and that by Sohn (22.6±12.2 [range: 12–58] months) [8]. This may explain the requirement for surgery, which could also be attributed to the higher prevalence of stentoliths [8]. The single stentolith encountered in our case series was managed endoscopically; therefore, surgery was not required in this case series.

Most of our patients stated that they were not informed of the need for biliary stent management. Trainee physicians involved in ERCP may forget to provide patients with such information [18]. In our centre, trainee physicians are in some cases responsible for providing patients with information on stent management. Six patients stated that they were expecting a telephone call from the hospital. In patients with ureteral stents, electronic reminders prevented forgotten stents [19,20] and could be similarly efficacious for biliary stents. In patients with choledocholithiasis, cholecystectomy is often performed after ERCP to prevent recurrent choledocholithiasis [21]. Some of our patients believed that their stent had been removed during cholecystectomy following ERCP and did not attend their follow-up ERCP appointment. Therefore, patients should be admitted to the Gastroenterology Department for stent removal after cholecystectomy.

We have begun to pay more attention to prevent FBS cases in our unit. The senior physician performing the ERCP procedure always instructs the patients’ relatives about subsequent management of an inserted biliary stent. A subsequent appointment for ERCP is arranged before discharge, and written details are provided to the patient and relatives. We emphasize the need for ERCP referral for patients undergoing cholecystectomy in which a biliary plastic stent was inserted before the surgery. As other preventive measure taken by our unit, all ERCP procedures are reviewed by a fellow at 3-month intervals, and the patients who do not present for removal of their stent in time are contacted. We also plan to implement an electronic reminder service for patients undergoing biliary stent insertion.

In conclusion, FBSs are potentially problematic and more common in older patients. Complications of FBS can be managed by endoscopic techniques. FBSs can be prevented by effective communication with the patient; reminder services could also be an option.

## Figures and Tables

**Table 1:** Demographic and clinical characteristics of study population.

**Baseline patient characteristics**	
Number of patients	959
Age, mean(SD), years	61.04 (±17.119)
Gender, male/female n(%)	486(51)/473(49)
ERCP procedures, n	1460
Stents inserted, n	574
Stent type (Benign/malignant condition)	
Plastic, n	270/217
Self-expandable metallic, n	17/70

**Characteristics of patients with FBS**	
Number of patients	44 (4 patients experienced two episodes of FBSs)
Age, mean(SD), years	71.23 (±12.165)
Gender, male/female	23/25
ERCP performed (M/F)	34 (17/17)
ERCP not performed (M/F)	14 (6/8)
Duration of stenting, months, median (range)	22.5(12–84 months)

**Causes of FBS**	n(%)
Uninformed patient	14(29.2)
Believed the stent had been removed	7 (14.6)
during cholecystectomy	
Expecting a telephone call	6(12.5)
Incompatible patient	6(12.5)
COVID-19-related	2(4.2)
No information available	13(27.1)

**Table 2:** Comparison of patients’ages between forgotten and timely removed stents.

	Stent not forgotten	FBS	p-value
**Number of patients**	155	44	
**Mean age(SD) years**	62.43(16.758)	71.23(12.165)	<0.001

**Table 3:** Stenting indications, clinical presentations, complications and endoscopic treatment of FBSs.

Parameter	n(%)

**Stenting indication**	
Irretrievable choledochal stones	40(83.3)
Benign biliary stricture	5(10.4)
Post-cholecystectomy biliary leak	3(6.2)

**Clinical presentation(ERCP+)**	
Cholangitis	22(64.7)
Jaundice	6(17.6)
Biliary pancreatitis	2(5.8)
Asymptomatic	4(11.7)

**FBS-related complications**	
Choledocholithiasis	27(79)
Proximal migration of stent	9(26.4)
Fracture of stent	3(8.8)
Giant stone	1(2.9)
Stentholith	1(2.9)

**Endoscopic treatment**	
Stent could not be retrieved, additional stent placed	5(14.7)
Stent retrieved by snare or basket	19(55.8)
Stent retrieved; new stent placed for large stones	9(26.4)
Stent retrieved; new stent placed for biliary stricture	1(2.9)
